# Efficacy and Potential Mechanism of Chinese Herbal Formula Chaihu Shugan Powder on Animal Models of Depression: A Systematic Review and Meta‐Analysis

**DOI:** 10.1111/cns.70375

**Published:** 2025-04-09

**Authors:** Lulu Wu, Yi Deng, Liyuan Yu, Weihang Peng, Ya Li, Yuchao Feng, Li Chen, Peiying Huang, Bojun Chen

**Affiliations:** ^1^ The Second Clinical School of Medicine Guangzhou University of Chinese Medicine Guangzhou China; ^2^ Clinical Research Team of Prevention and Treatment of Cardiac Emergencies With Traditional Chinese Medicine Guangdong Provincial Academy of Traditional Chinese Medicine Guangzhou China; ^3^ The Affiliated Brain Hospital of Guangzhou Medical University Guangzhou China; ^4^ Guangdong Provincial Hospital of Traditional Chinese Medicine Guangzhou China

**Keywords:** animal models, Chaihu Shugan powder, depression, mechanism, meta‐analysis

## Abstract

**Background:**

Chaihu Shugan powder (CSP) is used in the clinical treatment of depression. However, its clinical mechanisms remain elusive. While acknowledging the extensive animal research, there is a need for systematic meta‐analysis for comprehensive understanding. This study aims to evaluate the efficacy of CSP and its mechanisms in animal models of depression.

**Methods:**

Animal studies on the antidepressant effects of CSP were reviewed in databases including PubMed, Web of Science, Cochrane Library, Embase database, CNKI, VIP, WangFang database, and CBM published before March 12, 2024. The R Studio software was used for statistical analyses.

**Results:**

The present systematic review included 23 studies. The results revealed that CSP has a significant effect on behavior forced swimming test (FST) (SMD = −1.44, 95% CI: −1.87, −1.00); sucrose preference test (SPT) (SMD = 3.63, 95% CI: 1.87, 5.39), rearing numbers of open field test (OFT) (SMD = 1.46, 95% CI: 0.95, 1.96); crossing numbers of OFT (SMD = 2.15, 95% CI: 1.30, 3.01); and body weight (SMD = 2.16, 95% CI: 1.13, 3.18).

**Conclusion:**

In summary, this study revealed that CSP could treat depression symptoms in animals, which is associated with its anti‐inflammatory effects, regulation of the HPA axis, neurotrophic factors, and glucose metabolism.

## Graphical abstract

Chaihu Shugan powder is effective in improving depression symptoms in animals, which are related to anti‐inflammation, regulation of the HPA axis and neurotrophic factors, and glucose metabolism.

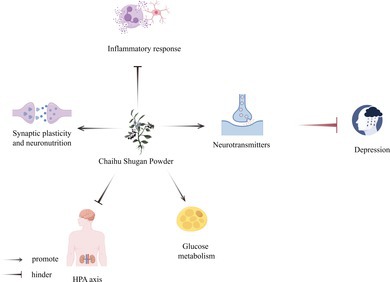

## 1 Introduction

Depression is a psychiatric condition characterized by various symptoms including low mood and loss of interest and pleasure, accompanied by anxiety, motor agitation, stupor, hallucinations, and delusions, among other psychotic symptoms [1]. Individuals may experience impaired self‐care and may be at risk for suicide in severe cases [2]. Research suggests that approximately 5% of adults globally suffer from depression annually, with a high prevalence reported in young individuals [3]. Furthermore, over 75% of individuals suffering from mental disorders remain untreated in low‐ to middle‐income countries [4]. Despite the availability of many antidepressants, there remains a significant unmet clinical need. Therefore, there is an urgent need for more clinical evidence to support the development and use of novel antidepressants [5].

Traditional Chinese Medicine (TCM) has stood out for its unique benefits and potential in treating depression, with reported clinical efficacy and mechanisms [6, 7]. Chaihu Shugan powder (CSP) is a popular formula documented in the Ming Dynasty medical compendium “Jingyue Quanshu”; it is traditionally used in TCM to resolve symptoms associated with liver qi stagnation. This formula has been broadly utilized to alleviate symptoms of depression [8], demonstrating safety and efficacy. CSP comprises Radix Bupleuri (Chaihu), *Citrus reticulata* (Chenpi), Paeoniae Radix Alba (Baishao), Aurantii Fructus (Zhiqiao), Cyperi Rhizoma (Xiangfu), Chuanxiong Rhizoma (Chuanxiong), and licorice (Gancao).

Previous investigations focused only on human studies [8], leaving out underlying mechanisms. Animal studies may elucidate the mechanisms underlying the treatment of depression; however, a comprehensive meta‐analysis is currently lacking. This has prevented a comprehensive understanding of the mechanisms and effects of CSP. To obtain valuable insights into its therapeutic potential, there is a need to simulate depressive symptoms in animal models and carry out tests to observe the effects of CSP, including forced swimming test (FST), sucrose preference test (SPT), and open field test (OFT). Therefore, this work aims to systematically assess the efficacy and mechanism of CSP in treating animal models of depression. This is geared towards improving the accuracy and dependability of experimental hypotheses as well as optimizing treatment approaches, hence providing robust therapeutic evidence and reference for future clinical research.

## 2 Methods

This study adhered to the Preferred Reporting Items for Systematic Reviews and Meta‐Analyses (PRISMA) statement guidelines for reporting this systematic review and meta‐analysis [9]. The protocol has been registered in PROSPERO (CRD42024567599).

### 2.1 Search Strategy

Two independent authors searched eight databases PubMed, Web of Science, Cochrane Library, Embase database, CNKI, VIP, WangFang database, and CBM to identify relevant animal studies from their inception to March 12, 2024. We searched the keywords terms as follows: (“Depression” OR “Depressive disorder”) AND (“Chaihu Shugan powder”, OR “Chaihu Shugan San”, OR “CSP” OR “CSS”) both in English and Chinese. There was no limitation on language or publication type.

### 2.2 Inclusion and Exclusion Criteria

The inclusion criteria included: (1) Animal experiments that assessed the effects of CSP focused on simple depression; (2) utilization of CSP as a standalone treatment in the intervention group; (3) adherence to the original herbal composition of CSP; (4) absence of intervention in the control group or administration of non‐functional substances including distilled water or saline; (5) employment of behavioral tests for depression as the primary outcome measures.

The exclusion criteria included: (1) duplicate articles, review articles, and clinical trials; (2) original text not available; (3) the original text with only charts and graphs, and data could not be obtained from the author.

### 2.3 Data Extraction

Two reviewers independently collected data. The extracted details included author, publication year, animal characteristics, reagent information, intervention details, major outcomes, and the corresponding *p*‐values. The standard deviation and mean values were primarily extracted as comparative metrics between the control and intervention groups. Discrepancies in study eligibility were resolved through consultation with a third reviewer. Original data from the included studies were acquired by contacting the authors via email in instances where only graphical representations were available. Discrepancies in outcome measurements were addressed through consensus. Only data with the highest dose were included in the analysis.

### 2.4 Risk of Bias Assessment

The quality of the studies included in the research was evaluated by two researchers using SYRCLE's risk of bias tool [10]. This assessment comprises 10 potential areas of bias, including selection, detection, performance, attrition, reporting biases, and other biases.

### 2.5 Statistical Analysis

Statistical analyses were conducted using the R Studio software. For continuous variables, the mean difference with 95% confidence intervals (95% CI) was used to assess the magnitude of the effect. Data analysis was carried out using the standardized mean difference (SMD) metric alongside 95% CI. The relevant results were aggregated using a random effects model to account for potential variability among studies. A significant difference between the intervention group and the control group was deemed present when the *p* < 0.05. The extent of heterogeneity among the results was quantified using the *I* ^2^ statistic. If *I* ^2^ > 50%, sensitivity or subgroup analyses were performed to sequentially examine the sources of heterogeneity and derive more stable conclusions.

## 3 Results

### 3.1 Study Inclusion

**FIGURE 1:**
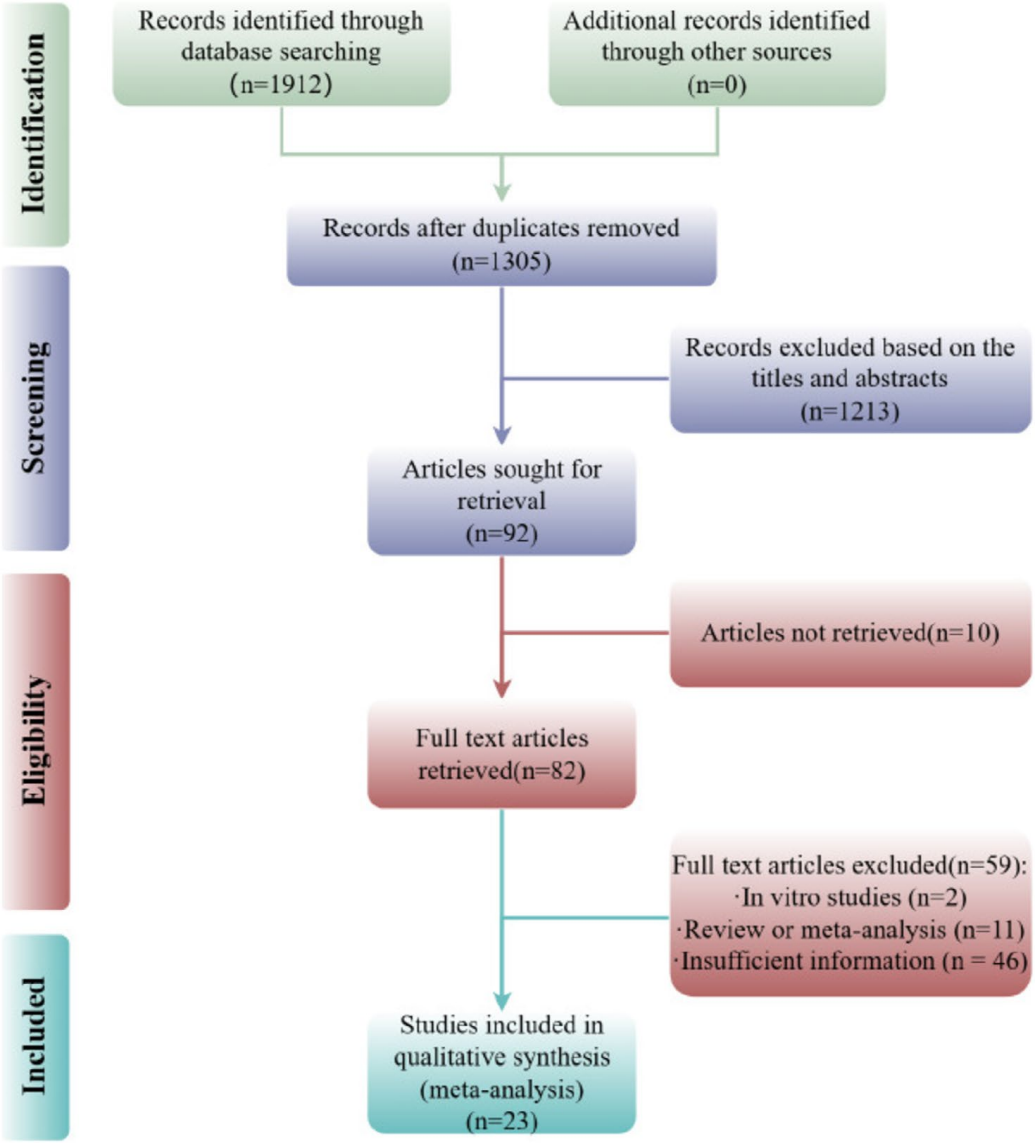
The flowchart of papers inclusion process.

In total, 1912 potentially relevant articles were identified from eight databases, including 50 from PubMed, 71 from Web of Science, 55 from Embase, 597 from CNKI, 625 from Wanfang, 441 from VIP, 12 from Cochrane Library, and 61 from CBM. A total of 1305 articles remained after removing 607 duplicates. Out of the 1305 records, 1223 studies did not meet inclusion criteria and were deleted after evaluating the titles and abstracts. Among these, 82 studies were excluded after screening the full‐text articles for various reasons, including not matching the original formula, not a single depression model, or active extract of CSP as the intervention group, review, clinical trials, cell culture study, and insufficient information. Eventually, 23 studies were included [11, 12, 13, 14, 15, 16, 17, 18, 19, 20, 21, 22, 23, 24, 25, 26, 27, 28, 29, 30, 31, 32, 33]. Figure 1 shows a graph detailing the search process.

### 3.2 Characteristics of the Included Studies

**FIGURE 2:**
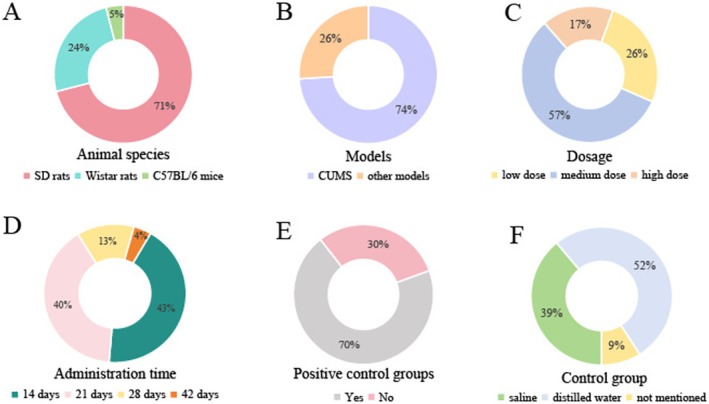
Included study characteristics. (A) Animal species; (B) models; (C) dosage; (D) administration time, (E) positive control groups, (F) intervention of control group.

Table 1 shows the basic characteristics of the 23 literatures. A total of 518 animals were included in this study, out of which 71% were Sprague–Dawley rats (368/518), 24% were Wistar rats (126/518), and 5% were C57BL/6 mice (24/518) (Figure 2A). The sample size for each study ranged from 16 to 30; 1 of the 23 studies used animals of half and half sexes, whereas the rest of the studies used male animals.

**TABLE 1:** Characteristics of included studies.

Author, publication year	Animal species, sex, number (E/M)	Body weight	Model	Intervention group	Control group	Administration time (days)	Positive control groups	Outcome indexes
Kim, 2005 [11]	SD rats (male, 10/10)	240–260 g	CMS, 21 days	2 g/kg/day	Distilled water	21	Imipramine	FST, *p* < 0.05
Li, 2014 [12]	SD rats (male, 10/10)	180–220 g	CUMS, 28 days	5.9 g/kg/day	Saline	14	Fluoxetine	OFT, *p* < 0.01 SPT *p* < 0.01
Teng, 2022 [13]	SD rats (male, 10/10)	180–200 g	CUMS, 28 days	11.25, 22.5, 45 g/kg/day	Saline	14	Venlafaxine	SPT, *p* < 0.05 FST, *p* < 0.05
Deng, 2011 [14]	SD rats (male, 10/10)	180–200 g	CUMS, 28 days	5.9 g/kg/day	Distilled water	14	Fluoxetine	OFT, *p* < 0.05
Dong, 2011 [15]	SD rats (male, 15/15)	360–380 g	CUMS, 21 days	1.4 g/kg/day	NM	21	Fluoxetine	SPT, *p* < 0.001 OFT, *p* < 0.001
Fan, 2019 [16]	Wistar rats (male, 10/10)	180–220 g	CUMS, 21 days	10.6 g/kg/day	Distilled water	21	Paroxetine	SPT, *p* < 0.01 FST, *p* < 0.01
Hu, 2010 [17]	SD rats (male, 10/10)	180–220 g	CUMS, 28 days	5.9 g/kg/day	Distilled water	14	Fluoxetine	OFT, *p* < 0.01
Liu, 2016 [18]	Wistar rats (male, 15/15)	180–220 g	CUMS, 22 days	10 g/kg/day	Saline	21	—	SPT, *p* < 0.05
Liu, 2020 [19]	SD rats (male and female, 12/12)	223.2 ± 4.5 g	CUMS, 28 days	10/20/30 g/kg/day	Saline	14	Fluoxetine	FST, *p* < 0.05
Liu, 2013 [20]	Wistar rats (male, 10/10)	180–210 g	CUMS, 21 days	10 g/kg/day	NM	21	—	SPT, *p* < 0.05 TST, *p* < 0.05
Qiu, 2014 [21]	SD rats (male, 15/15)	180–220 g	CUMS, 28 days	5.9 g/kg/day	Distilled water	28	Fluoxetine	OFT, *p* < 0.01 SPT, *p* < 0.05
Wang, 2014 [22]	SD rats (male, 15/15)	180–220 g	ip 0.5 mg/kg reserpine for 14 days	5, 10 g/kg/day	Distilled water	14	Fluoxetine	OFT, *p* < 0.05
Wang, 2012 [23]	SD rats (male, 12/12)	180–220 g	ip 0.5 mg/kg reserpine for 14 days	5, 10 g/kg/day	Saline	14	Fluoxetine	OFT, *p* < 0.05
Yang, 2023 [24]	SD rats (male, 10/10)	180–220 g	ip 0.5 mg/kg LPS for 14 days	21 mg/kg/day	Saline	21	—	SPT, *p* < 0.001
Yang, 2019 [25]	Wistar rats (male, 10/10)	200–220 g	CUMS, 28 days	0.6, 1.2 g/kg/day	Distilled water	14	Fluoxetine	SPT, *p* < 0.05
Yu, 2014 [26]	SD rats (male, 15/15)	280–320 g	CUMS, 21 days	7.875 g/kg/day	Saline	21	—	OFT, *p* < 0.05
Zhang, 2020 [27]	SD rats (male, 10/10)	200–220 g	CUMS, 56 days	2.8 g/kg/day	Saline	28	Citalopram	SPT, *p* < 0.05 FST, *p* < 0.05
Wang, 2015 [28]	SD rats (male, 12/12)	160–200 g	ip 0.5 mg/kg reserpine for 14 days	5, 10 g/kg/day	Distilled water	14	Fluoxetine	OFT, *p* < 0.05
Wu, 2018 [29]	Wistar rats (male, 8/8)	180–210 g	CUMS, 21 days	6.3 g/kg/day	Distilled water	21	Paroxetine	SPT, *p* < 0.05 TST, *p* < 0.05
Wang, 2023 [30]	Wistar rats (male, 10/10)	170–190 g	Combine CUMS and Solitary feeding, 21 days	5.2, 10.4 g/kg/day	Saline	21	—	SPT, *p* < 0.05 OFT, *p* < 0.05
Xiao, 2021 [31]	SD rats (male, 10/10)	180–220 g	CUMS, 56 days	2.8 g/kg/day	Distilled water	28	Citalopram	OFT, *p* < 0.05 FST, *p* < 0.05 SPT, *p* < 0.05
Cao, 2013 [32]	SD rats (male, 8/8)	180–200 g	CUMS, 28 days	5.9 g/kg/day	Distilled water	14	—	OFT, *p* < 0.05 SPT, *p* < 0.05
Zhang, 2022 [33]	C57BL/6 mice (male, 12/12)	—	CUMS, 42 days	19.5 g/kg/day	Distilled water	42	—	FST, *p* < 0.01 SPT, *p* < 0.05 TST, *p* < 0.05

Abbreviations: CMS, chronic mild stress; CUMS, chronic unpredictable mild stress; E, experiment; FST, forced swim test; LPS, lipopolysaccharide; M, model; NM, not mentioned; OFT, open field test; SPT, sucrose preference test; TST, tail suspension test.

### 3.3 Experimental Models

Five different types of animal models were incorporated in the included studies, among which 17 (74%) used the chronic unpredicted mild stress (CUMS) model; the remaining 6 (26%) used other models like Lipopolysaccharide or reserpine injection, chronic mild stress (CMS) model, and a combination of CUMS and solitary feeding (Figure 2B).

All studies had the animals administered with CSP, with a dose ranging from 0.02 to 45 g/kg, and divided into three groups (low dose: less than 5 g/kg, medium dose: 5–10 g/kg, high dose: greater than 10 g/kg). Dosage distribution was as follows: low dose 26% (6/23), medium dose 57% (13/23), high dose 17% (4/23) (Figure 2C). Eight studies included multiple dose groups.

Administration was primarily through oral gavage (100%, 23/23), with treatment cycles spanning 14 days (43%, 10/23), 21 days (40%, 9/23), 28 days (13%, 3/23), and 42 days (4%, 1/23) (Figure 2D).

In total, 70% (16/23) of the studies included in the analysis incorporated positive control groups using various antidepressants including fluoxetine, imipramine, venlafaxine, paroxetine, and citalopram, whereas the remaining six studies did not (Figure 2E). The majority of control groups received either saline (39%, 9/23) or distilled water (52%, 12/23), with a minority of studies failing to specify the control substance used (9%, 2/23) (Figure 2F).

### 3.4 Quality Evaluation

**FIGURE 3:**
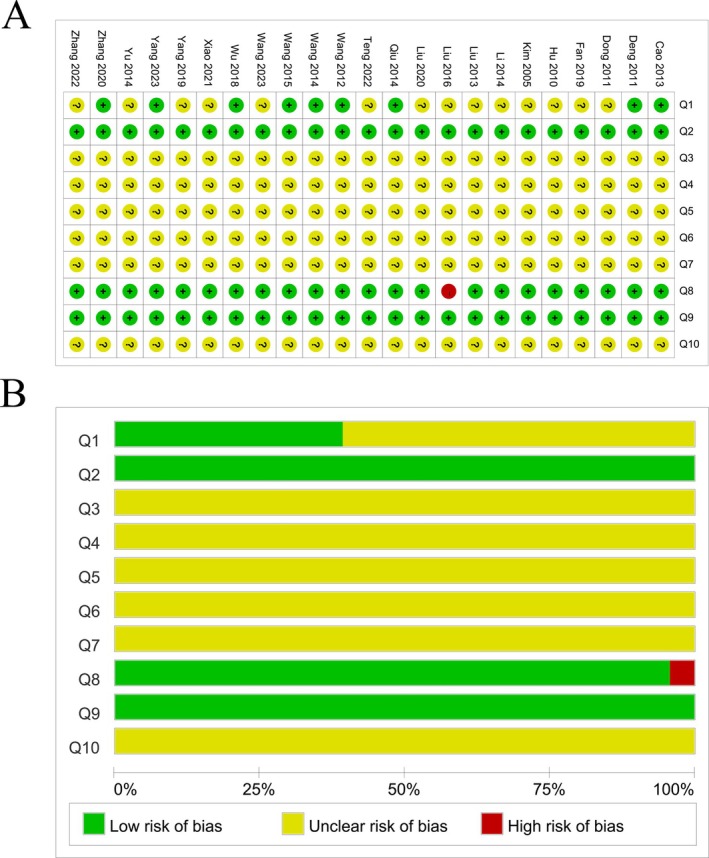
Risk of bias and quality of included studies. (A) Risk of bias summary. (B) Risk of bias graph. (1) Whether the allocation sequence is produced or applied adequately; (2) whether the baseline of each group is the same; (3) allocation to hide whether sufficient; (4) whether the animals were randomly placed during the experiment; (5) whether to blind researchers; (6) whether the animals were randomly selected in the result evaluation; (7) whether the result evaluator adopts the blind method; (8) incomplete data are reported; (9) whether the study report is independent of the report of selective results; (10) whether there is no other bias.

Overall, the risk of bias assessment revealed a predominance of “unclear” ratings, suggesting widespread under‐reporting of key items and the potential for an indeterminate risk of bias. Regarding selective bias (Q1–Q3), 23 papers referenced randomization of groups, with only 9 [14, 21, 22, 23, 24, 27, 28, 29, 32] mentioned providing details on the grouping method, leaving the remaining 15 unidentifiable. Across all studies, baseline reporting was consistent, with no mention of allocation concealment. Implementation bias (Q4–Q5) was not explicitly addressed in any of the studies. Furthermore, none of the studies detailed the random selection of animals during outcome evaluation or whether blinding techniques were employed by evaluators (Q6–Q7). Incomplete data in a study by Liu et al. [18] were not sufficiently addressed, causing an increased risk of bias (Q8). Reporting bias was deemed low in all studies, whereas the presence of other biases remained uncertain (Q10) (Figure 3).

### 3.5 Body Weight and Behavioral Change

Body weight, OFT, FST, and SPT are commonly used assessments in depression models. Herein, 11 studies used body weight as an indicator, 7 studies employed the FST, 16 studies incorporated the SPT, and 12 studies adopted the OFT. Among these studies, 11 evaluated the crossing times of animals in the OFT, whereas 12 assessed the rearing times of animals during the OFT. Other behaviors including tail suspension test [20, 29, 33] and Morris water maze [18] were measured in several studies.

#### 3.5.1 Effects of CSP on Body Weight

**FIGURE 4:**
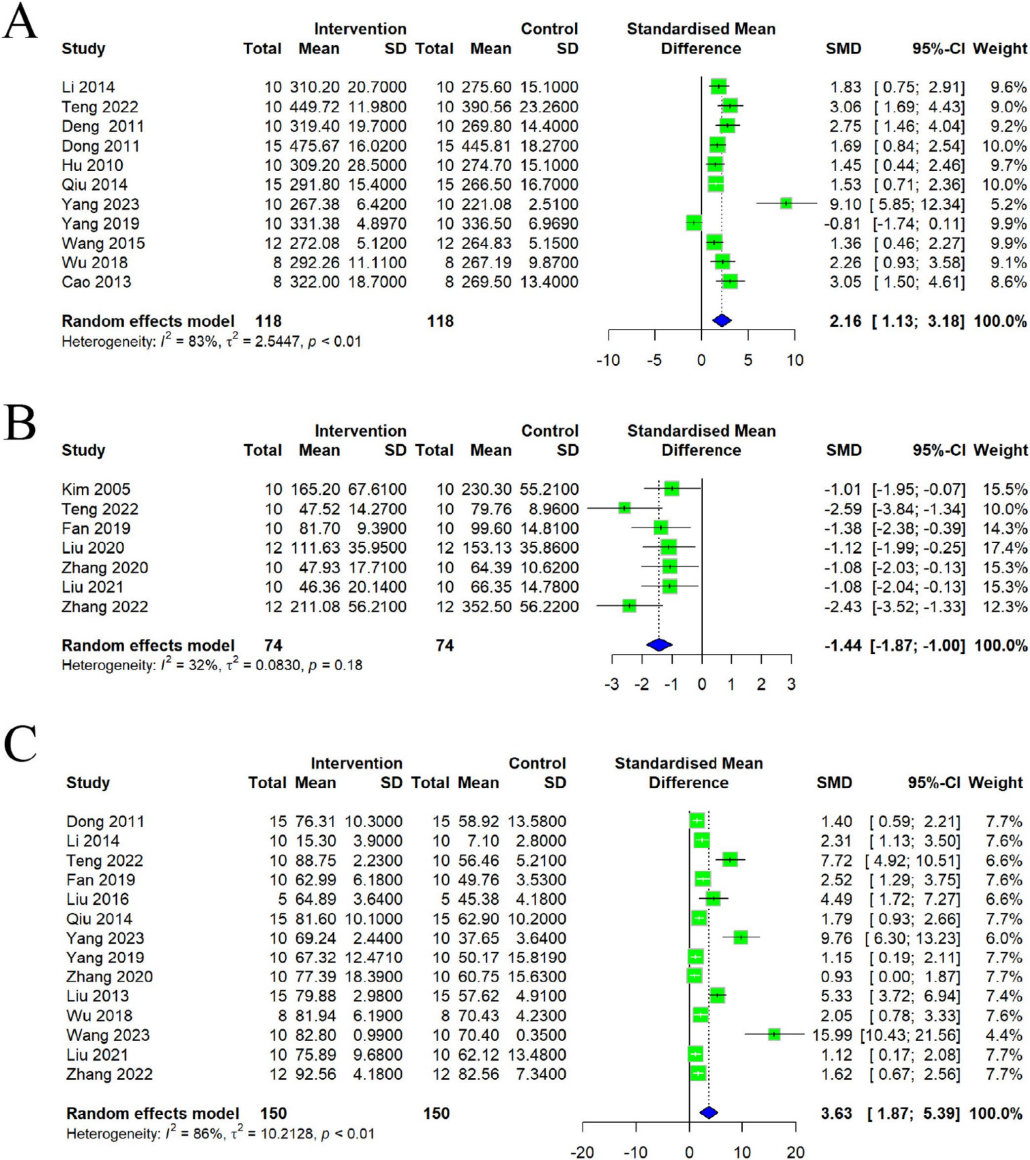
Forest plot of (A) body weight, (B) FST, and (C) SPT.

A total of 11 studies indicated a significant decrease in body weight after the depression modeling process; CSP caused a significant increase. Regression analysis revealed that the disparities among models could account for the observed heterogeneity (*p* < 0.01, *I* ^2^ = 83%) (Figure 4A).

#### 3.5.2 Effects of CSP on Behavioral Change

##### 3.5.2.1 Effects of CSP on FST

FST duration among depression‐affected animals was significantly reduced by CSP, as shown in seven studies. As illustrated in Figure 4B, the results indicated a stable result of FST, with minimal heterogeneity across the included studies (*p* = 0.18, *I* ^2^ = 32%).

##### 3.5.2.2 Effects of CSP on SPT

Further, 14 studies investigated SPT in evaluating anhedonia in depression. The CSP intervention group showed a significant increase in SPT compared to the model group (Figure 4C). A significant amount of heterogeneity was found in the random effects model (*p* < 0.01, *I* ^2^ = 86%).

##### 3.5.2.3 Effects of CSP on OFT

**FIGURE 5:**
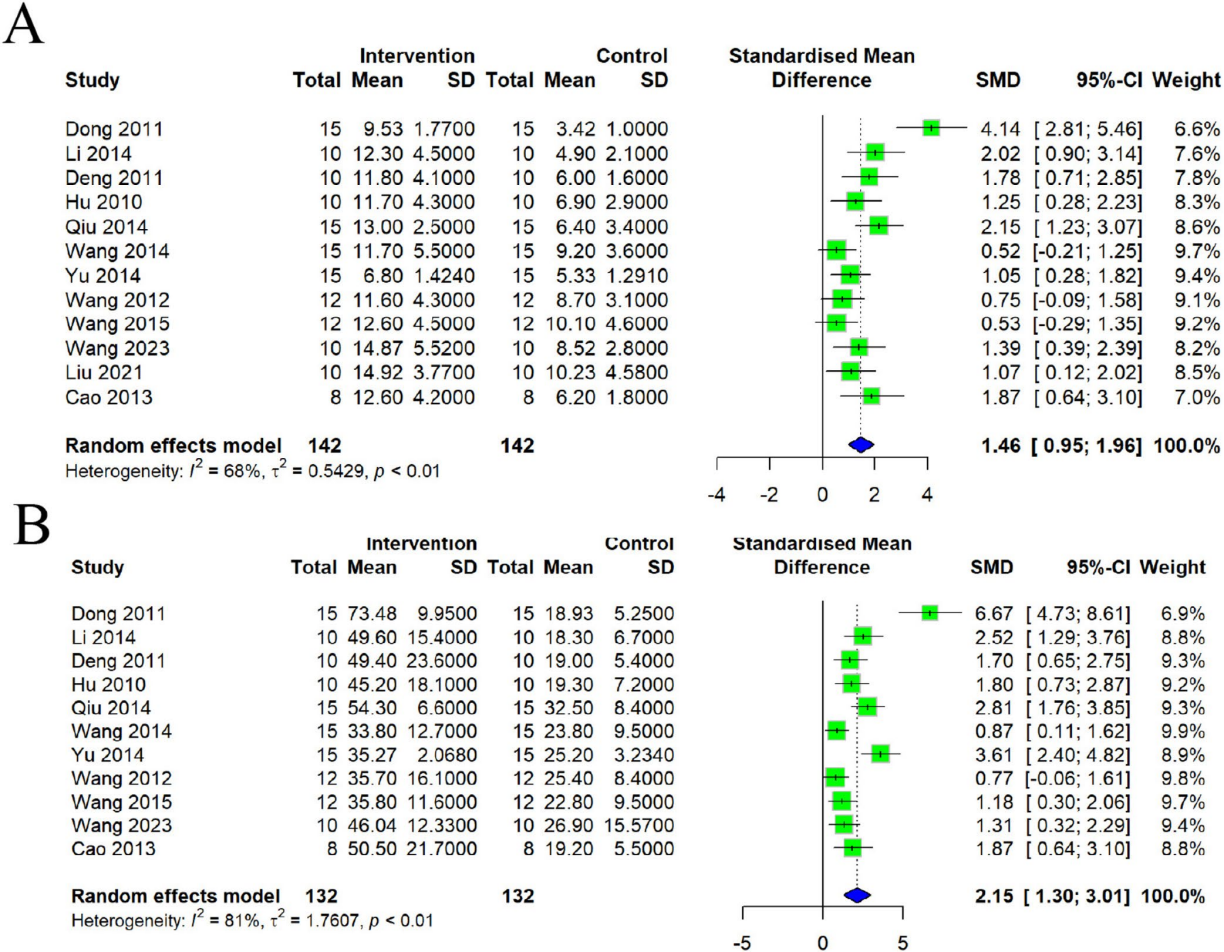
Forest plot of (A) rearing numbers and (B) crossing numbers of OFT.

A significant increase in rearing and crossing behaviors was observed in the open field test after CSP intervention. Data on rearing times in the OFT were obtained from 12 studies. Meta‐analysis showed a significant increase in rearing numbers; however, the results were heterogeneous (*I* ^2^ = 68%, *p* < 0.01), requiring a random effects model (Figure 5A).

A total of 11 studies were conducted to collect data on crossing numbers in the OFT, revealing a statistically significant increase in crossing numbers with CSP intervention. Nonetheless, a high level of heterogeneity was observed (*I* ^2^ = 81%, *p* < 0.01) (Figure 5B), necessitating the use of a random effects model for assessment.

#### 3.5.3 Sensitivity Analysis

**FIGURE 6:**
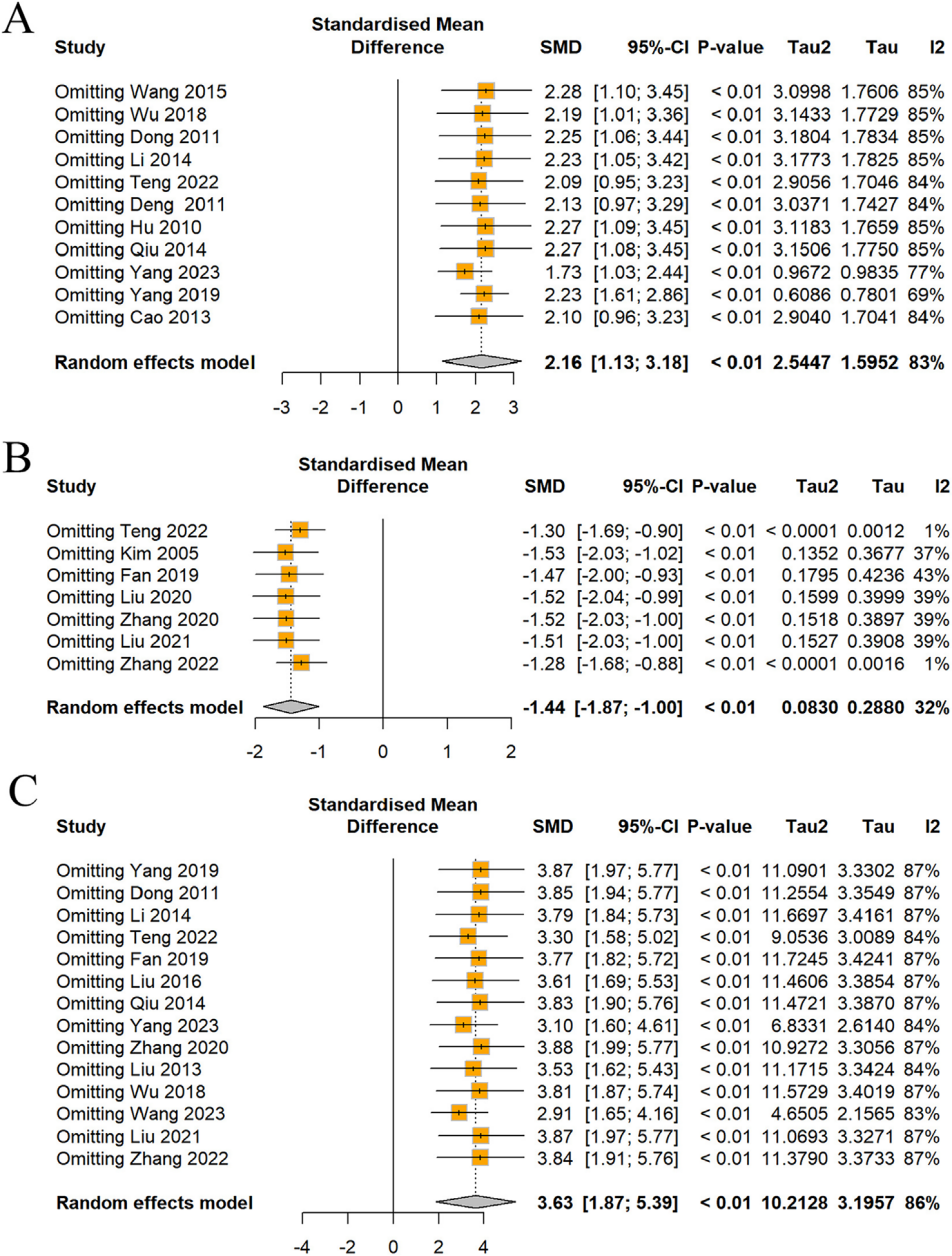
Sensitivity analysis of (A) body weight, (B) FST, and (C) SPT.

**FIGURE 7:**
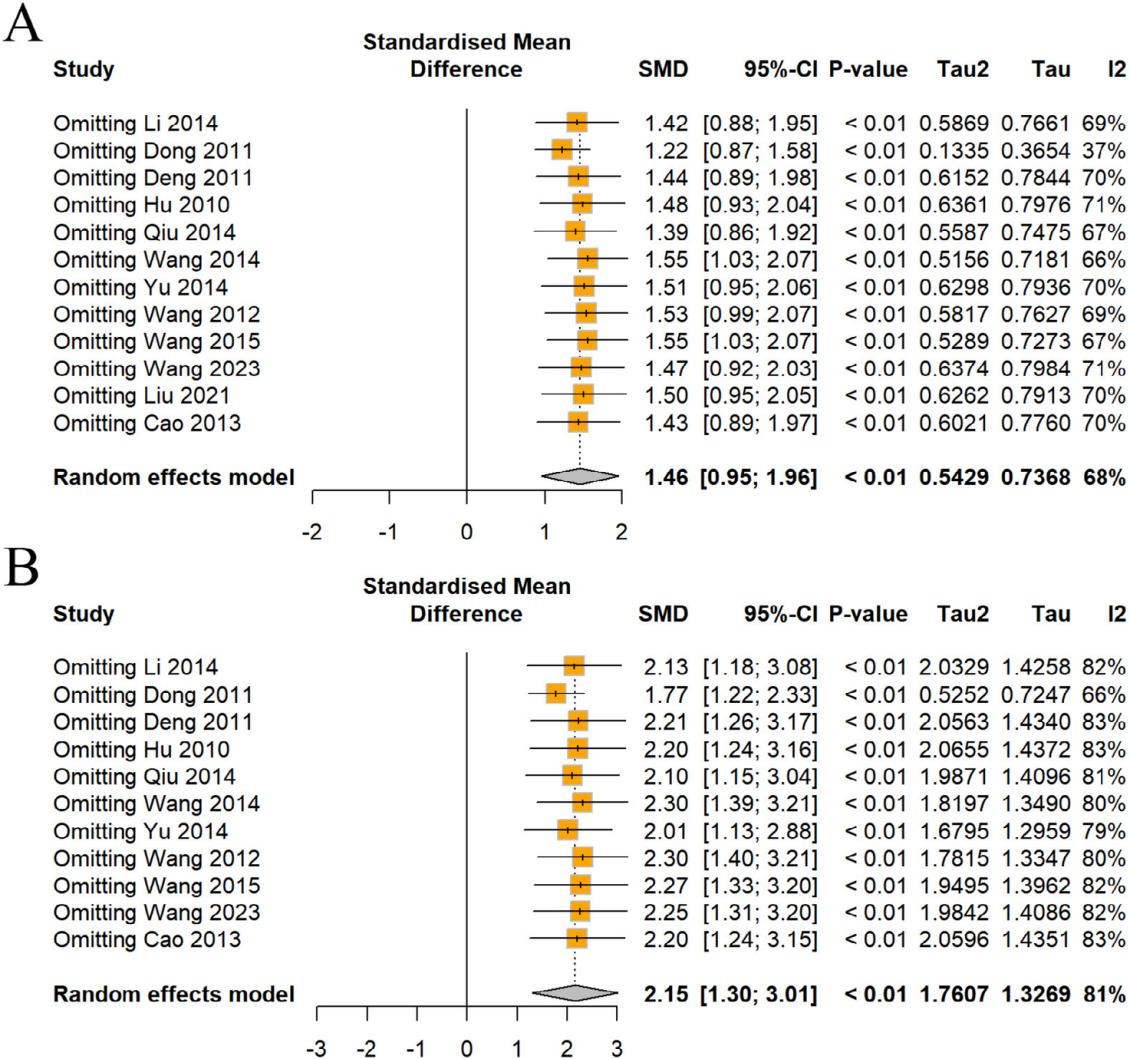
Sensitivity analysis of (A) rearing numbers and (B) crossing numbers of OFT.

Despite sequentially excluding individual studies, the heterogeneity remained substantial for body weight and behavioral indicators (Figures 6 and 7). However, an intriguing pattern emerged, while the heterogeneity in FST scores remained consistently low across all exclusions (Figure 6B); the heterogeneity in rearing numbers experienced a notable decrease from 68% to 37% solely upon excluding the study by Dong et al. [15] (Figure 7A). This suggests that the study by Dong may be a key contributor to the observed heterogeneity in rearing numbers, despite the precise source of this variability being elusive.

#### 3.5.4 Subgroup Analysis

**FIGURE 8:**
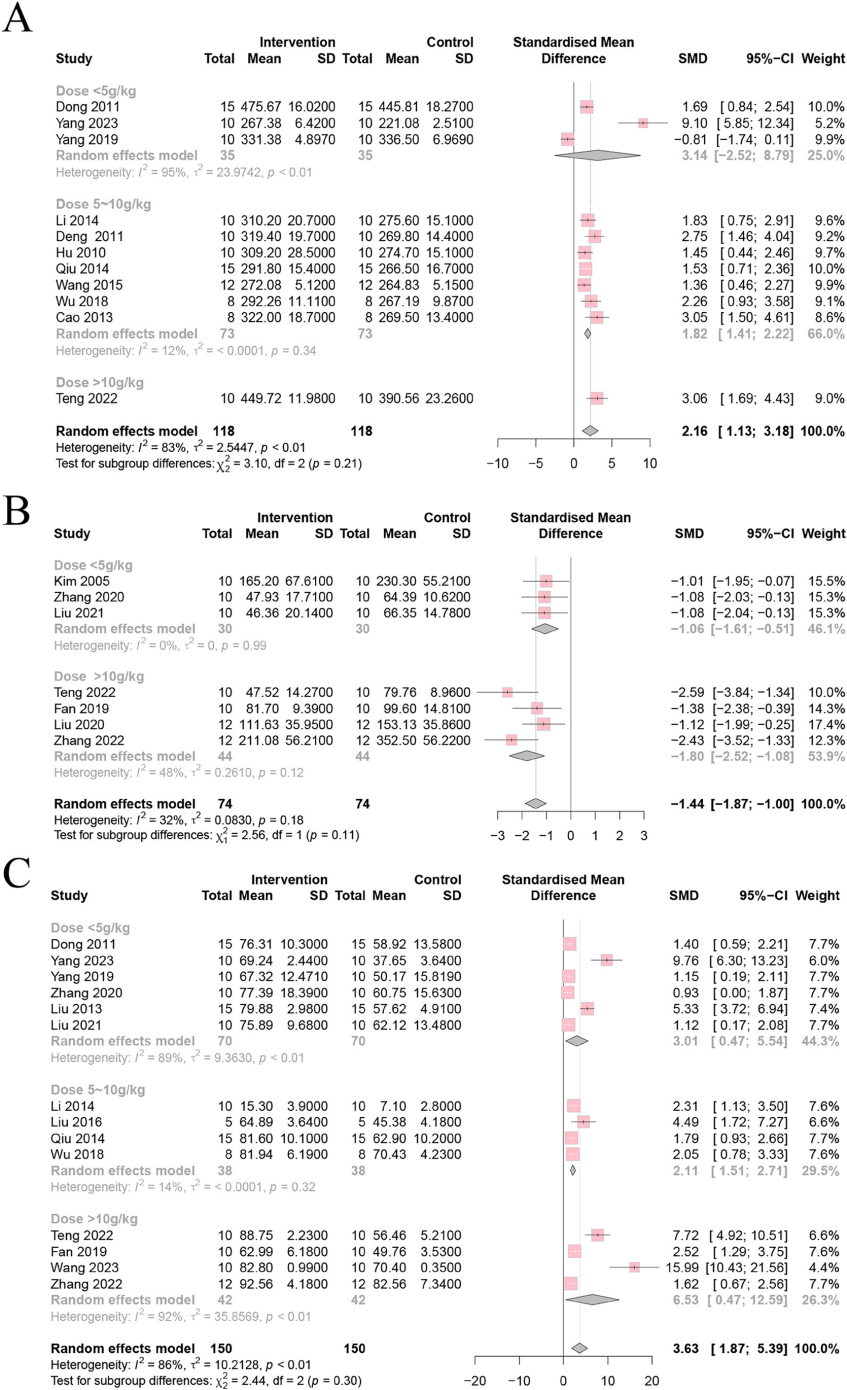
Forest plot subgroup comparison according to the range of the dosage of Chaihu Shugan powder in the (A) body weight, (B) FST, and (C) SPT.

**FIGURE 9:**
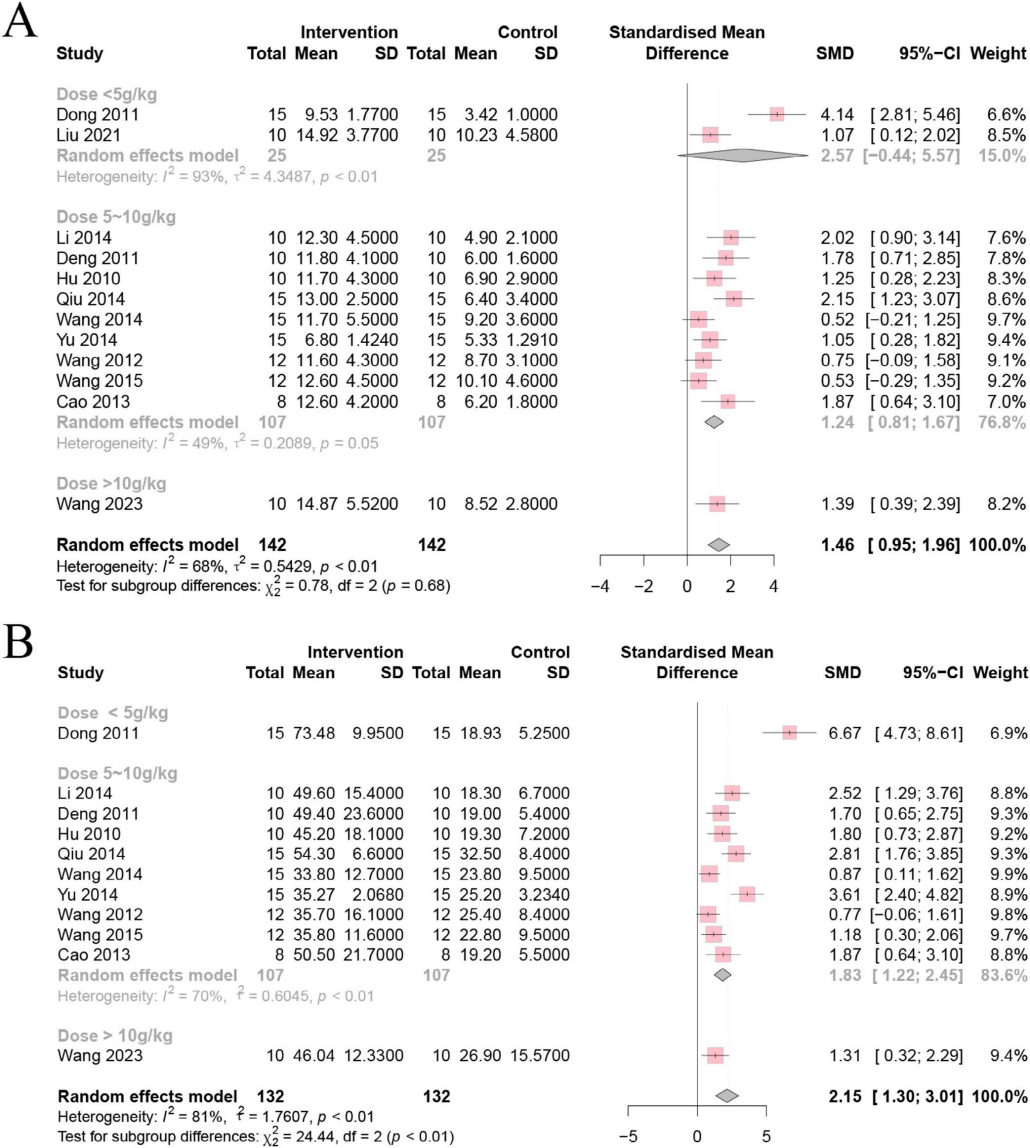
Forest plot subgroup comparison according to the range of the dosage of Chaihu Shugan powder in the (A) rearing numbers and (B) crossing numbers of OFT.

Subgroup analysis was carried out after regression and sensitivity analyses. First, subgroup analysis was conducted via the dosage range of CSP. Weight loss and behavioral changes were improved with different dosages. The results showed that a medium dose of CSP had the best effects on body weight (*p* = 0.34, *I* ^2^ = 12%) (Figure 8A), whereas low dose results were inconsistent; only one study used a high dose. FST heterogeneity at different doses was not statistically significant (Figure 8B). The heterogeneity of medium dose for SPT (*p* = 0.32, *I* ^2^ = 14%) (Figure 8C) and rearing numbers (Figure 9A) was significantly reduced; this indicates that the difference in dose was one of the sources of heterogeneity. Contrary to this, the dose did not influence the heterogeneity of crossing numbers (*p* = 0.05, *I* ^2^ = 49%) (Figure 9B).

**FIGURE 10:**
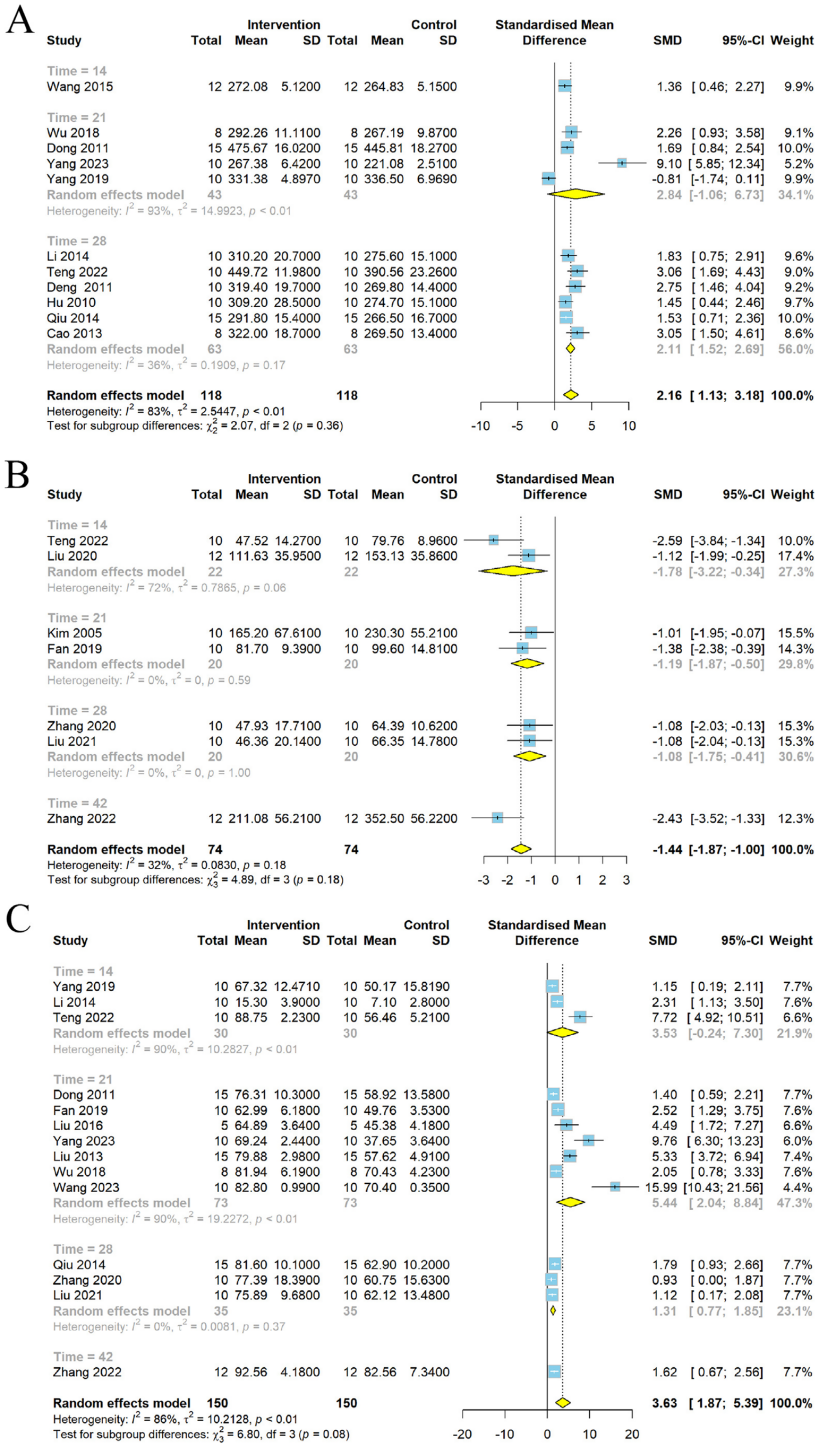
Forest plot subgroup comparison according to the intervention time of Chaihu Shugan powder in the (A) body weight, (B) FST, and (C) SPT.

**FIGURE 11:**
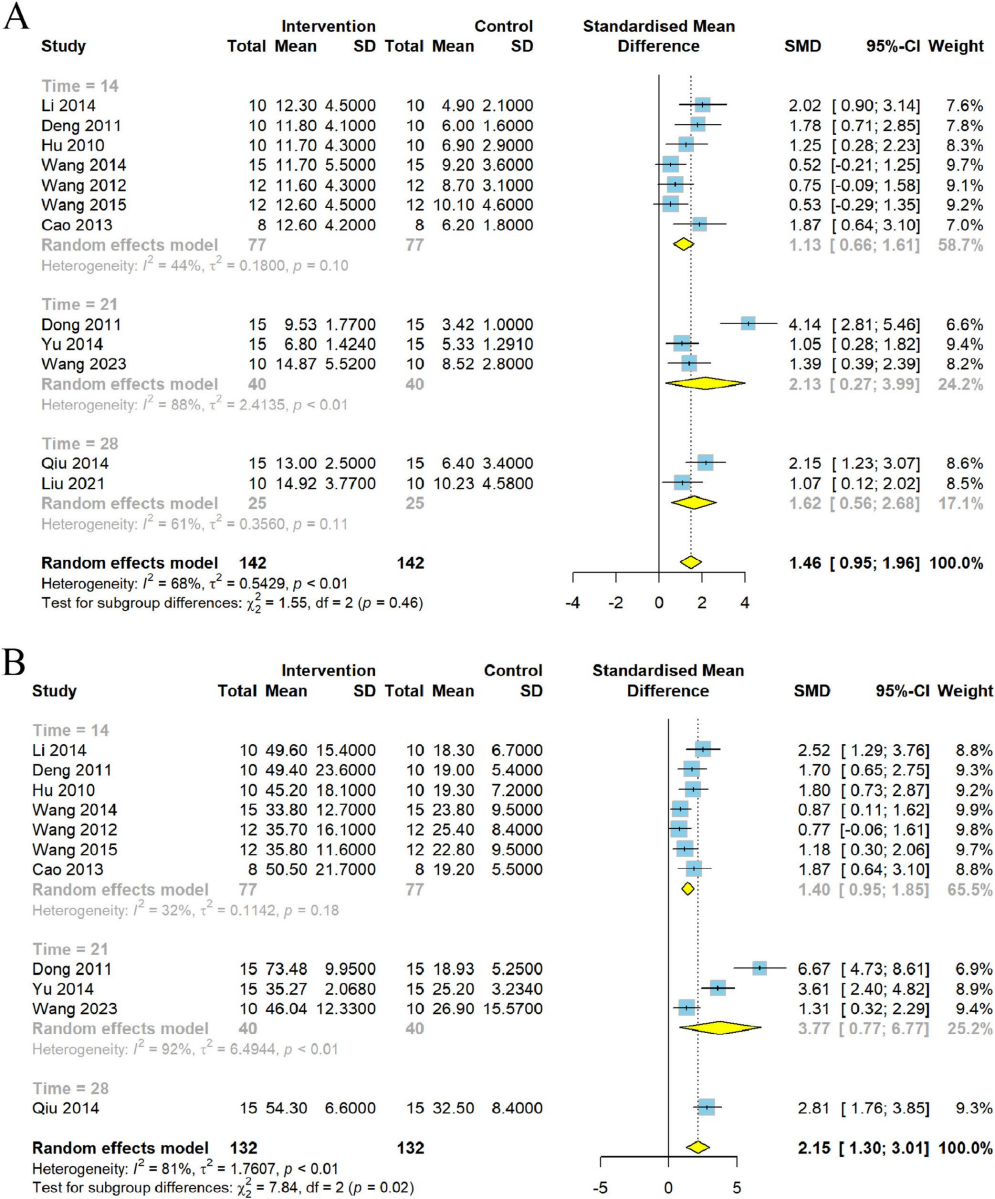
Forest plot subgroup comparison according to the intervention time of Chaihu Shugan powder in the (A) rearing numbers and (B) crossing numbers of OFT.

According to a subgroup analysis of intervention time, different intervention times also caused different degrees of weight loss and behavior change. The 28‐day group showed significantly reduced heterogeneity of body weight (*p* = 0.17, *I* ^2^ = 36%) (Figure 10A) and SPT (*p* = 0.37, *I* ^2^ = 0%) (Figure 10C), whereas the 14‐day group showed reduced heterogeneity of rearing numbers (*p* = 0.10, *I* ^2^ = 44%) (Figure 11A) and crossing numbers (*p* = 0.18, *I* ^2^ = 32%) (Figure 11B). The effects of CSP on FST were stable in the 21‐ and 28‐day groups (Figure 10B).

**FIGURE 12:**
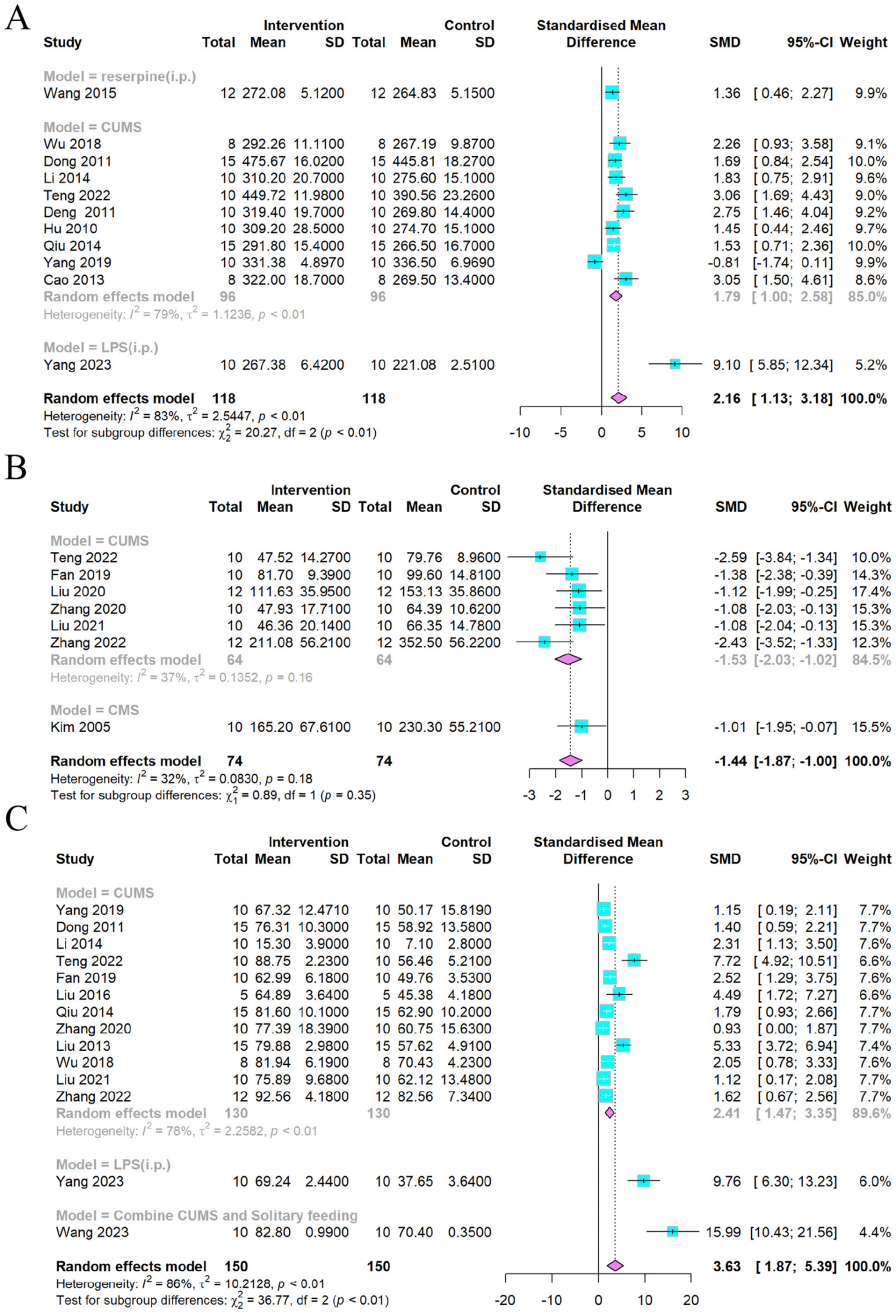
Forest plot subgroup comparison according to the different models in the (A) body weight, (B) FST, and (C) SPT.

**FIGURE 13:**
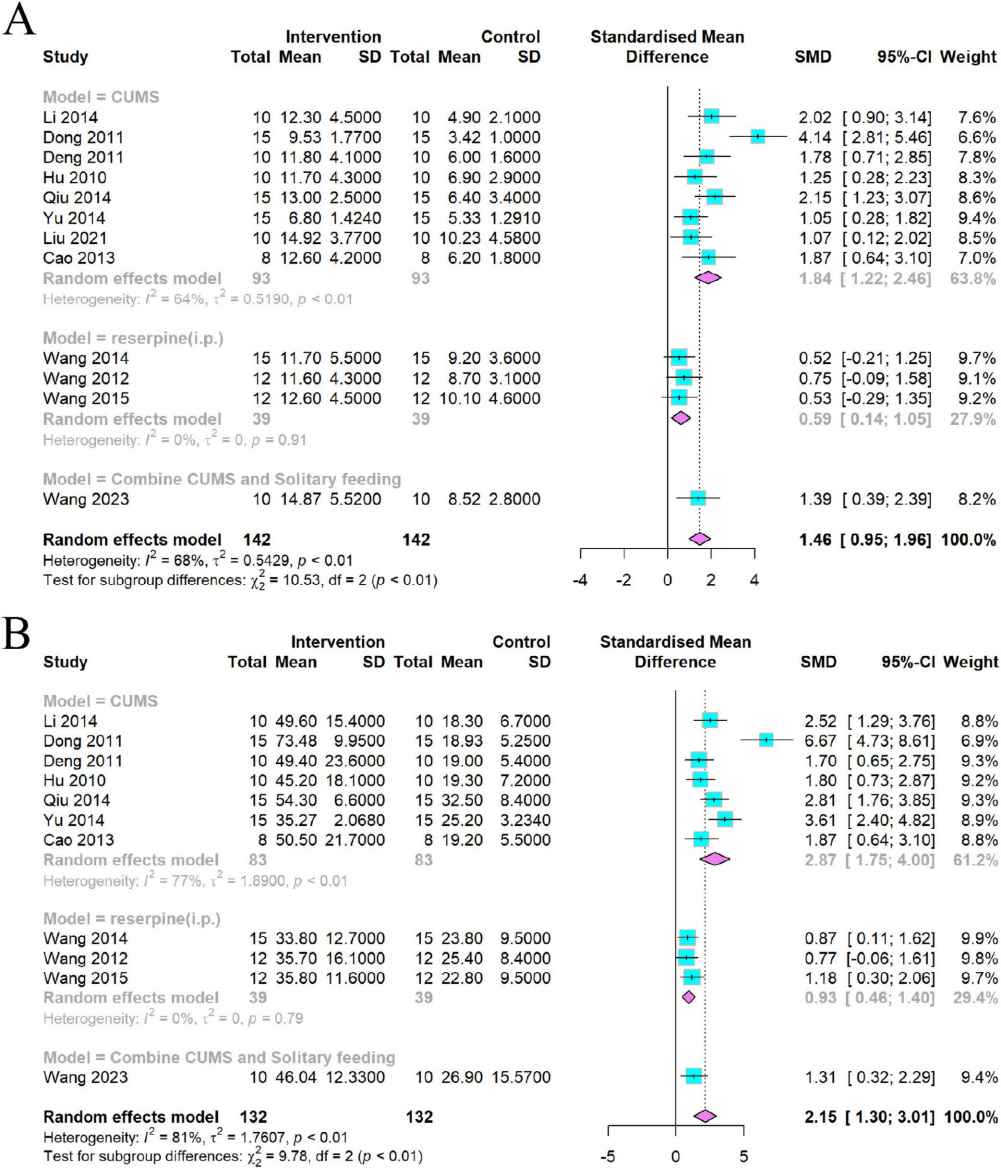
Forest plot subgroup comparison according to the different models in the (A) rearing numbers and (B) crossing numbers of OFT.

In addition to the dose and duration of the intervention, we also examined whether different models affected the effect of the CSP on each indicator. In terms of body weight (Figure 12A) and SPT (Figure 12C), different models did not significantly change the results, and the CUMS model was stable for FST (Figure 12B). The reserpine‐induced depression model for rearing numbers (Figure 13A) and crossing numbers (Figure 13B) indicated low heterogeneity and stable results.

#### 3.5.5 Publication Bias

**FIGURE 14:**
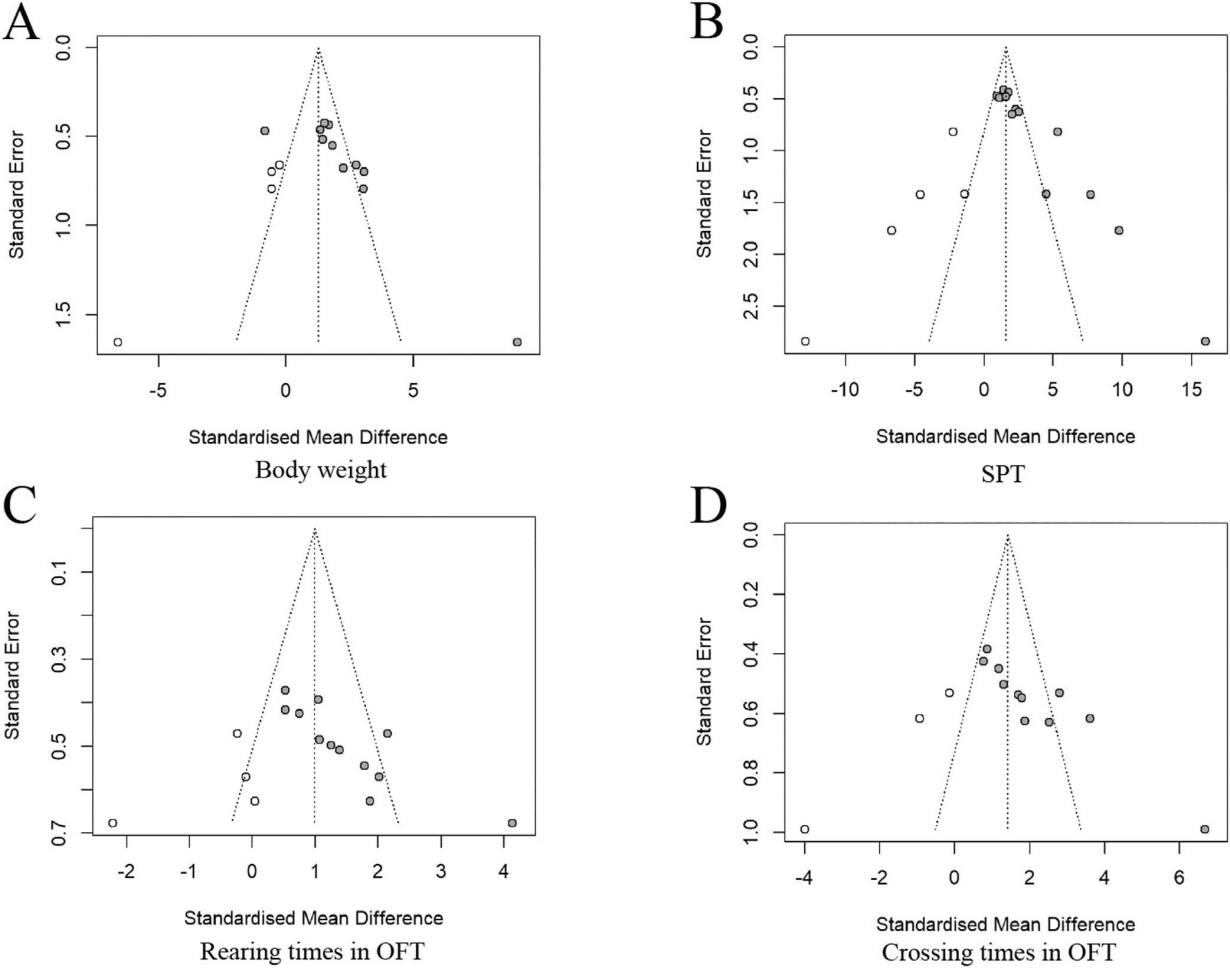
Publication bias of Chaihu Shugan powder. (A) The funnel plots of the body weight; (B) the funnel plots of the SPT; (C) the funnel plots of the rearing numbers in the OFT; and (D) the funnel plots of the crossing numbers in the OFT.

A low risk of publication bias was found in the funnel plot of the meta‐analysis. A low publication bias was observed in the effect of CSP on body weight and SPT (Figure 14A,B), as well as a symmetry in the funnel plot of OFT (rearing numbers and crossing numbers) (Figure 14C,D).

#### 3.5.6 Possible Mechanism of CSP in Depression

Table 2 summarizes the possible mechanisms of CSP treatment for depression, primarily encompassing the regulation of neurotransmitters and the hypothalamic pituitary adrenal (HPA) axis, anti‐inflammatory effects, modulation of neurotrophic factor synthesis, and glucose metabolism in the brain.

**TABLE 2:** Possible mechanism of the treatment of Chaihu Shugan powder for depression.

Author, publication year	Tissue	Proposed mechanism	Molecular changes
Li, 2014 [12]	Hippocampus	Regulating SAPK/JNK pathways	↓: JNK, p‐JNK
Teng, 2022 [13]	Hippocampus	Promoting PI3K/Akt pathways	↑: NE, 5‐HT ↑: p‐PI3K/PI3K, p‐Akt/Akt
Deng, 2011 [14]	Frontal cortex and amygdala	Regulation of the BDNF and neuroprotective effect	↑: BDNF, TrkB
Dong, 2011 [15]	Hippocampus	Adjust the choline nervous system and monoclonal nervous system	↑: MAO, AChE
Fan, 2019 [16]	Hippocampus	1. Inhibition of inflammation 2. Regulation of the synaptic plasticity 3. Regulation of BDNF/TrkB/NF‐κB pathways	↓: TNF‐α, NF‐κB ↑: TrkB, PSD95, GLuR1
Hu, 2010 [17]	Plasma	Regulation of the HPA axis	↓: CRH, ACTH
Liu, 2016 [18]	Hippocampus	Regulation of MAP‐2 to maintain neuronal morphology and synaptic plasticity	↑: MAP‐2
Liu, 2016 [19]	Hippocampus, and plasma	1. Regulation of HPA axis 2. Regulation of BDNF/TrkB pathways	↓: CRH, ACTH ↑: E_2_, DA, NE, 5‐HT, GHR ↑: BDNF, TrkB
Liu, 2013 [20]	Hippocampus	Regulation of 5‐HT receptor	↑: 5‐HT, 5‐HT1A
Qiu, 2014 [21]	Hippocampus	Improving ERK to regulate adult hippocampal neurogenesis	↑: ERK
Wang, 2014 [22]	Hippocampus	Regulation of central monoamine	↑: DA, NE, 5‐HT
Yang, 2023 [24]	Hippocampus	1. Inhibition of inflammation 2. Regulation of AKT1/CREB1/FOS pathways	↓: IL‐6 ↑: AKT1, CREB1, FOS
Yang, 2019 [25]	Hippocampus, hypothalamus, and hypophysis	1. Regulation of neuro‐immune function 2. Inhibition of inflammation 3. Regulation of HPA axis	↑: DA, NE, IL‐10, IL‐1β, IL‐6, TNF‐α, IDO, GR, Rab4a ↓: 5‐HT, CRH, CORT, ACTH, Lta4h, Cyp2s1
Yu, 2014 [26]	Hippocampus, hypothalamus, and plasma	Regulation of the HPA axis by GR	↓: ACTH, CORT, CRH↑: GR
Zhang, 2020 [27]	Prefrontal cortex	Promoting cAMP/CREB/BDNF pathways and neuroprotective effects	↑: cAMP, CREB, BDNF
Wang, 2015 [28]	Hippocampus	Regulation of the 5‐HT system by THP2	↑: 5‐HT, THP2
Wang, 2023 [30]	Brain tissue	Ameliorating glucose uptake competition	↑: glucose metabolic rate
Xiao, 2021 [31]	Prefrontal cortex	1. Inhibition of inflammation 2. Regulation of SIRT1/NF‐κB pathways	↓: TNF‐α, IL‐1β, NF‐κB, Caspase3 ↑: SIRT1, IκBα
Cao, 2013 [32]	Hippocampus	Regulating miR‐125a, and miR‐182 to improve the plasticity of hippocampus neurons	↑: miR‐125a ↓: miR‐182
Zhang, 2022 [33]	Hippocampus	Promoting angiogenesis and neurogenesis in the hippocampus according to SIRT1/FOXO1pathway	↑: SIRT1, FOXO1, BDNF, VEGFA

Abbreviations: 5‐HT, serotonin; 5‐HT1A, serotonin‐1A; AChE, acetylcholinesterase; ACTH, adrenocorticotropic hormone; Akt, protein kinase B; BDNF, brain‐derived neurotrophic factor; cAMP, cyclic adenosine monophosphate; CORT, corticosterone; CREB1, cyclic adenosine monophosphate response element‐binding protein 1; CRH, corticotropin releasing hormone; Cyp2s1, cytochrome P450 family 2; DA, dopamine; E2, estradiol; ERK, extracellular regulated protein kinase; FOS, c‐Fos protein; FOXO1, forkhead box protein 01; GHR, ghrelin; GLuR1, glutamate receptor; GR, glucocorticoid receptor; GRα, glucocorticoid receptor alpha; HPA, hypothalamic–pituitary–adrenal; IDO, indoleamine 2,3‐dioxygenase; IL‐10, interleukin 10; IL‐1β, interleukin‐1 beta; IL‐6, interleukin 6; IκBα, NF‐kappa‐B inhibitor alpha; JNK, c‐Jun N‐terminal kinase; Lta4h, leukotriene A4 hydrolase; MAO, monoamine oxidase; MAP‐2, microtubule‐associated protein 2; NE, norepinephrine; NF‐κB, nuclear factor kappa‐B; PI3K, phosphatidylinositol‐4,5‐bisphosphate 3‐kinase; PSD95, postsynaptic density protein 95; Rab4a, ras‐related protein Rab.4A; SAPK, stress activated protein kinase; SIRT1, sirtuin 1; THP2, tryptophan hydroxylase 2; TNF‐α, tumor necrosis factor‐alpha; TrkB, tropomyosin receptor kinase B; VEGFA, vascular endothelial growth factor A.

Studies have shown that CSP can increase the levels of monoamine neurotransmitters including 5‐hydroxytryptamine (5‐HT), norepinephrine (NE), and dopamine (DA) [13, 18, 19, 22, 25, 28]. It also attenuates stress responses by reducing the levels of HPA axis hormones like corticotropin‐releasing hormone (CRH), adrenocorticotropic hormone (ACTH), and corticosterone (CORT) [17, 18, 25, 26]. Additionally, CSP indirectly modulates the HPA axis by enhancing the expression of microtubule‐associated protein 2 (MAP‐2), thereby improving neuronal plasticity [18].

CSP exerts anti‐inflammatory properties by decreasing IL‐6 and TNF‐α levels, thus mitigating HPA axis hyperactivity [26] and enhancing hippocampal neuronal plasticity through the upregulation of tropomyosin receptor kinase B (TRKB) [16]. CSP stimulates sirtuin 1 (SIRT1) and inhibits the activity of nuclear factor kappa‐B (NF‐κB) [31], thereby reducing inflammatory responses.

Regarding brain‐derived neurotrophic factor (BDNF), CSP regulates neural growth by enhancing BDNF expression and promoting vascular endothelial growth factor (VEGF) synthesis [14, 16, 27, 33, 34]. Notably, Zhang et al. [33] discovered that CSP boosted hippocampal angiogenesis and neurogenesis via the SIRT1/FOXO1 pathway to promote the expression of VEGFA. Furthermore, CSP promotes neurological recovery by improving intracerebral glucose metabolism and enhancing glucose uptake [30].

## 4 Discussion

This meta‐analysis sought to primarily investigate the treatment effects of CSP on various symptoms of depression and elucidate its underlying mechanisms of action. Depression, which is a prevalent mental health condition, can be caused by various underlying factors and manifests itself with weight loss, consistent with alterations in weight observed in animal models of depression [35]. Our findings indicate that CSP mitigates weight loss and restores weight gain in the model group. The outcomes in both the medium‐dose CSP and 28‐day intervention groups were stable, whereas the effects of different models on the results were not significant, that is, the source of heterogeneity is not from the differences in models, but the doses and intervention time. Therefore, when applied for longer intervention periods, CSP may achieve more sustained effects, especially in chronic depression models where the long‐term effect of the medication may contribute significantly to weight restoration.

The FST is a commonly used behavioral assay in research settings that replicates a stressful and inescapable environment for animals. When subjected to this test, animals initially display vigorous struggling behavior in an attempt to escape the confined swimming environment. However, as they realize that escape is impossible, they finally enter a state of “behavioral despair,” indicated by a cessation of struggling and swimming. One of the primary indicators of depressive behavior in the FST is prolonged immobility time [36]. CSP significantly decreases FST immobility time, implying that CSP may ameliorate negative emotions, promote positive emotions, and alleviate depressive symptoms to some extent. Low heterogeneity suggests a high consistency among different experimental results, which may be attributed to the adoption of a more standardized experimental design, similar animal models, and matching intervention dosages and durations. This demonstrates that CSP had stable effects on depression models. The SPT is a behavioral assessment method tailored to the innate preference for sweet flavors in animals. When animals develop depressive‐like behaviors, their preference for sugar water diminishes [37]. Meta‐analytic findings reveal that depressed animal models exhibit reduced sucrose consumption, indicating a suppression of happiness. Intriguingly, CSP significantly improves SPT scores, suggesting that it can restore pleasurable behaviors and alleviate depressive symptoms to a considerable extent. The high heterogeneity inspired subgroup analysis, which suggested a similar outcome to body weight, with stable results in both medium‐dose and 28‐day intervention groups but not associated with model differences. The most stable effects were achieved with moderate dosages and longer intervention durations, suggesting that CSP's efficacy in modulating appetite and reward mechanisms in depression models depends on dosage and intervention duration. OFT is a well‐established method for evaluating autonomous behavior, exploratory tendencies, and anxiety levels of experimental animals in unfamiliar environments, providing valuable insights into their emotional state and response to novelty [38]. CSP significantly increases both the crossing and rearing numbers, indicating its potential to restore a desire to explore a new environment. The heterogeneity of rearing numbers was reduced upon removing the study by Dong et al. [15]. Subgroup analysis showed stable medium‐dose CSP, but not significant for the crossing numbers. A 14‐day intervention timing showed a relatively low heterogeneity; as for the model, the results of reserpine‐induced depression were stable. The high heterogeneity of OFT may be due to the differences between the dose and intervention time of CSP compared to the model. These findings highlight potential differences in medication responses between acute and chronic animal models, necessitating model‐specific adjustments in dosage and intervention duration to optimize therapeutic outcomes. These findings show that CSP ameliorates depressive behaviors; however, the underlying mechanisms remain to be fully elucidated. Based on the findings from the included studies, we propose that the antidepressant effects of CSP may be mediated through several key mechanisms, including the suppression of inflammation, modulation of the HPA axis and neuroprotective processes, and the regulation of neurotransmitter function and glucose metabolism. Researchers commonly hypothesize that depression is associated with inadequate activity of monoamine neurotransmitters, including 5‐HT, NE, and DA in the brain. CSP also increased the monoamine transmitter expression in our study. Similar to the mechanism of the existing antidepressant drugs, CSP may alleviate symptoms by increasing the production of these neurotransmitters, balancing monoamines generation, and improving neurotransmission. The HPA axis hyperfunction is a contributing factor to depression. The hypothalamus secretes CRH, stimulating ACTH release from the pituitary and CORT from the adrenal cortex, causing sympathetic responses. GR downregulation in the hippocampus disrupts HPA axis negative feedback, causing sustained activation and depression [39]. Our findings showed that CSP administration suppressed hyperactivity within the HPA axis. Unlike conventional antidepressants, CSP may modulate the HPA axis more gently, reducing the risk of side effects from prolonged pharmacological treatment. Besides the HPA axis, immune responses have also been involved in depression [40]. A previous meta‐analysis has shown that increased microglial activity induces increased levels of TNF, interleukin‐8 (IL‐8), and IL‐6 in cerebrospinal fluid (CSF) and brain tissue of patients with depression [41]. Furthermore, both IL‐6 and TNF can increase the permeability of the blood‐brain barrier (BBB) [42, 43]. The compromised BBB allows inflammatory agents to infiltrate, causing neuroinflammation and eventually depression. CSP may mitigate depression by reducing inflammation, protecting the BBB, and improving the CNS inflammatory microenvironment. Through these mechanisms, CSP potentially modulates neuroendocrine and immune systems via multiple pathways, offering a holistic therapeutic approach.

Specifically, the expression level of BDNF in depressed patients is often diminished, specifically in the hippocampus, potentially linked to a decline in neural plasticity [44]. Besides its role in angiogenesis, VEGF also regulates neurogenesis and synaptic plasticity [45]. CSP can alleviate depression by increasing BDNF levels, improving nerve nutrition and angiogenesis, and promoting hippocampal nerve nutrition. This finding demonstrates the comprehensive effects of TCM in the treatment of depression, extending beyond mere neuromodulation to include enhanced neuronal regeneration and restoration processes.

Glucose metabolism plays a critical role in neurodegenerative diseases due to metabolic abnormalities causing energy production defects, altering neurotransmitter release, and suppressing emotional and cognitive functions [46]. Our findings demonstrate that CSP can significantly improve glucose metabolism. This beneficial effect may contribute to the therapeutic efficacy of CSP in depression by mitigating the detrimental effect of impaired glucose metabolism on neuronal function and mitigating associated neurological dysfunction.

In summary, CSP induces diverse effects on animal models of depression. Firstly, the observed effects of CSP may vary across different animal models of depression due to model‐dependent factors such as the duration of the model and potential differences in drug metabolism and efficacy. In addition, we found that the effect was dose‐dependent, with the dosage being a crucial factor influencing therapeutic outcomes. The therapeutic efficacy of CSP may be limited by both insufficient and excessive dosing. While low doses may be ineffective, excessively high doses can increase the risk of side effects and potentially compromise treatment stability. Optimal therapeutic outcomes are typically observed within a moderate dosing range. Moreover, the duration of intervention influences the efficacy of CSP. Extended intervention periods, which extend to 28 days, may exert a more sustained treatment effect, particularly in chronic depression models where long‐term intervention stabilizes the behavior of animals. Future research should investigate the impact of different dosing regimens and intervention durations on the efficacy of CSP in a variety of depression models. Furthermore, clinical trials are warranted to evaluate the clinical feasibility and effectiveness of CSP as a potential therapeutic agent for depression.

Overall, the efficacy of CSP in animal models of depression is noteworthy, yet this work has several limitations Firstly, a significant number of primary studies originate from the same country, which may introduce various forms of bias. Specifically, regional restrictions could lead to an overly homogenous sample. If most studies are conducted in a single country, the geographical and cultural backgrounds of the samples may lack diversity. This uniformity might limit the generalizability of the results, as experimental environments, research practices, and designs in other regions could differ, thereby influencing the external validity of the conclusions. In the second place, discrepancies in experimental design and reporting standards constitute a non‐negligible issue. Studies from the same country often adhere to similar experimental protocols and reporting standards, which may obscure certain deviations. For instance, the variables considered crucial in other regions might not receive adequate attention or control in these studies, thereby impacting the accuracy and reliability of the findings. Thirdly, the cultural dependency on herbal medicines can also introduce bias. As a traditional Chinese medicine formula, the use and research of Chaihu Shugan San are primarily concentrated in Asian countries including China, Korea, and Japan. This cultural reliance might hinder the research conclusions from fully indicating global perspectives on its efficacy across different cultural backgrounds, limiting its widespread adoption. Furthermore, publication bias represents a potential concern. Studies originating from specific regions may have a higher likelihood of being published, while those with less favorable results often get overlooked or remain unpublished, causing selection bias and affecting the objectivity of the overall research findings. Lastly, sample bias arises from the possibility that sample sources are influenced by specific demographic characteristics, health statuses, and socioeconomic conditions of the country. A particular nation might have a different disease burden, healthcare system, and health behaviors, potentially resulting in a selected sample that is not representative of the global population or other countries. Such sample bias can compromise the external validity of the results.

## 5 Conclusion

In conclusion, the etiology of depression is complex and diverse; hence, we recommend the use of CSP to effectively alleviate depression. CSP holds potential in the treatment of depression by modulating these interrelated pathways and provides a promising direction for future research. Nonetheless, considering its limitations, future research should be more diversified and rigorous to ensure its efficacy is widely validated across different regions and cultural backgrounds.

## Author Contributions

Lulu Wu, Yi Deng, Liyuan Yu, Weihang Peng, Peiying Huang, and Bojun Chen conceived the study. Lulu Wu and Liyuan Yu collected the data. Lulu Wu performed a meta‐analysis. Yuchao Feng, Li Chen, and Yi Deng prepared the figures and tables. Lulu Wu drafted the manuscript. Peiying Huang, Weihang Peng, and Ya Li revised it. All authors discussed and approved the final manuscript.

## Conflicts of Interest

The authors declare no conflicts of interest.

## Data Availability

Research data are not shared.
